# Debiasing or regularisation? Two interpretations of the concept of ‘true preference’ in behavioural economics

**DOI:** 10.1007/s11238-022-09876-x

**Published:** 2022-02-28

**Authors:** Robert Sugden

**Affiliations:** School of Economics, University of East Anglia, Norwich, NR4 &TJ UK

**Keywords:** Regularisation, True preference, Behavioural bias, Behavioural welfare economics, Stated preference

## Abstract

I reconsider Bleichrodt, Pinto Prades and Wakker’s (BPW) 2001 paper about eliciting utility measures from stated preference surveys. That paper pioneers a method that is now widely used in behavioural economics to correct individuals’ ‘biases’ and to recover their ‘true preferences’. However, BPW propose this method as way of dealing with inconsistent responses to stated preference surveys, in contrast to more recent applications which aim to help individuals to avoid supposed mistakes in their private choices. I argue that the concepts of true preference and bias are empirically ungrounded, but that BPW’s approach can be interpreted as not invoking those concepts. By ‘regularising’ preferences revealed in actual choice, this approach constructs measures of individual welfare that are broadly aligned with actual preferences and consistent with normative standards of rationality that are appropriate for public decision-making. Public decision-makers’ normative judgements are made explicit, rather than being disguised as apparently empirical claims about true preferences.

## Introduction

One of the biggest problems in behavioural economics is to find a method of normative analysis that can be used even when individuals’ revealed preferences contravene the rationality axioms of neoclassical economics. Before behavioural economics took off in the 1980s and 1990s, there was a firmly established consensus about how to do normative analysis in economics. That analysis was interpreted as a study of welfare, and there was general agreement about the basic principles of welfare economics. Those principles rested on the assumption that, with respect to whatever outcomes were the subject of economic analysis, each individual’s preferences were *complete* (i.e., every pair of outcomes was ranked by strict preference or indifference), *consistent* (i.e., compatible with standard rationality axioms), *context-independent* (i.e., independent of welfare-irrelevant properties of particular decision situations), and *revealed in choice*. A person’s preferences were interpreted as providing (or defining) an ordinal measure of her welfare. However, we now know from the findings of behavioural research that the preferences revealed in people’s actual choices show systematic patterns that contravene consistency and context-independence and which, although susceptible to psychological explanation, cannot plausibly be interpreted as relevant for assessments of welfare. If normative analysis in economics is to be reconciled with behavioural findings, something in the previous consensus has to be given up.

This paper is concerned with one broad strategy for tackling this problem, characterised by Infante et al. (2016) as *behavioural welfare economics*. This approach retains the assumption that individuals have complete, consistent and context-independent preferences, and treats those preferences as indicators of welfare. What is given up is the assumption that those preferences are reliably revealed in choice. Instead, some concept of *true* (or ‘underlying’ or ‘latent’) preference is invoked. Completeness, consistency, context-independence and welfare relevance are attributed to true preferences, but actual choices are explained as resulting from the combined effects of true preferences and ‘biases’ or ‘errors’. In this paper, I compare two different ways of interpreting the concept of true preference.

It is generally held that the founding contributions to behavioural welfare economics were made in 2003 in two remarkably similar papers by American behavioural economists and legal scholars, proposing ‘asymmetric paternalism’ (Camerer et al., 2003) and ‘libertarian paternalism’ (Sunstein & Thaler, 2003). However, my focus will be on a paper by Bleichrodt, Pinto-Prades and Wakker (BPW) which advocated a form of behavioural welfare economics two years before the American papers (Bleichrodt et al., 2001). One significant feature of the earlier paper is that, unlike the later two, it proposes an operational method by which an analyst can identify individuals’ true preferences. Variants of that method have subsequently been advocated by other behavioural economists (e.g., Beshears et al., 2008: 1790–1791; Kőszegi & Rabin, 2008), and BPW’s pioneering work has not received the full credit it deserves. In this paper, however, I am more concerned with another important difference between BPW’s paper and its successors.

It is now often forgotten how much early work in behavioural economics was carried out in response to problems uncovered by *stated preference* studies. Such studies use survey methods to elicit individuals’ preferences for non-marketed goods, typically for use in cost–benefit or cost-effectiveness analysis. Common applications include the elicitation of individuals’ valuations of changes in the provision of environmental public goods, changes in health states, and changes in accident risks. At a time when most of the data used by economists was highly aggregated, stated preference surveys were unusual in generating individual-level preference data that were capable of revealing direct violations of rationality axioms. Since the whole project of stated-preference research presupposed the existence of consistent and context-independent preferences, the discovery of systematic inconsistencies in the data (for example, disparities between willingness-to-pay and willingness-to-accept, part-whole disparities, and the dependence of survey responses on the scale on which those responses are elicited) created serious problems. Initially, many economists attributed these inconsistencies to supposed defects in the survey instruments used, or to the fact that stated-preference surveys necessarily use hypothetical questions. An early research programme in behavioural economics investigated whether the same anomalies occurred in controlled experiments in which subjects made real choices over familiar consumer goods. It found the same qualitative effects (e.g., Bateman et al., 1997a, 1997b). One response to this problem was to look for ways of retrieving consistent ‘true’ preferences from survey responses. BPW’s work belongs to that project.

In contrast, Camerer et al. (2003) and Sunstein and Thaler (2003) start from the premise that, as a result of psychological mechanisms identified by behavioural economics, individuals make choices that are contrary to their best interests. Their papers are written as justifications of paternalistic interventions designed to steer people away from such choices. Thus, Camerer et al. say: ‘Recent research in behavioral economics has identified a variety of decision-making errors that may expand the scope of paternalistic regulation. To the extent that the errors identified by behavioral research lead people not to behave in their own best interests, paternalism may prove useful’ (pp. 1211–1212). Similarly, Sunstein and Thaler say: ‘Drawing on some well-established findings in behavioral economics and cognitive psychology, we emphasize the possibility that in some cases individuals make inferior decisions in terms of their own welfare’. In such cases, they recommend ‘private and public planners’ to ‘self-consciously attempt to move people in welfare-promoting directions’ (p. 1162). For these authors, the distinction between revealed preference and true preference is conceptualised in terms of the presence or absence of *error* on the part of the individuals whom the proposed interventions are to *help*. ‘Error’ and ‘helping’ are recurrent tropes in both papers. Evidence of inconsistency or context-dependence in an individual’s preferences is treated as evidence of error and of a consequent need for help.

There is a fundamental difference here. The premises of BPW’s project are not paternalistic. Their running examples are about cost-effectiveness in a European-style health care system in which health care is free at the point of delivery and is financed through taxation or social insurance. In such a system, it is a fact of life that decisions have to be made about the allocation of resources between classes of patients with different medical conditions and between alternative treatments for given conditions. If, in making those decisions, policy-makers believe that they should be guided by information about citizens’ preferences, that is the opposite of paternalism. The problem is to find a way of extracting relevant information from inconsistent survey responses. Ultimately, it is the policy-makers, not the citizens, who are in need of help. In the current paper, I explore some of the implications of that difference by examining BPW’s formal analysis and the arguments by which they justify it.

The first aim of my paper, and the subject of Sects. 1 to 4, is interpretative—to elucidate BPW’s own understanding of the method they propose. I consider two alternative interpretations. The *debiasing* interpretation assimilates BPW’s method to the form of behavioural welfare economics proposed by Camerer et al. and Sunstein and Thaler in their 2003 papers. It treats BPW’s method as an attempt to reconstruct individuals’ true preferences by removing the effects of errors that those individuals make when taking decisions for themselves, or when reporting their own preferences. In contrast, the *regularisation* interpretation, first discussed by Infante et al. (2016), treats BPW’s method as a means of constructing indices of individual welfare that are broadly consistent with individuals’ revealed preferences and that satisfy principles of rationality that are judged to be appropriate for public decision-making.^1^ If individuals’ revealed preferences contravene standard rationality conditions, that shows that some regularisation is needed in order to create useful welfare indices, but it is not treated as evidence that those individuals are making errors. I find textual support for each of these interpretations in different parts of BPW’s paper, but argue that the most consistent interpretation of their method is in terms of regularisation.

In Sect. 5, I characterise a general method of regularisation that is consistent with (and, I suggest, implicit in) BPW’s work. In the final section I argue that this method is coherent and defensible, and that, unlike the debiasing approach to behavioural welfare economics, it is not vulnerable to the criticism that it invokes psychologically ungrounded concepts of true preference and bias. But I must emphasise that the content of my paper is not a proposal *from me* about how welfare economics should be adapted in the light of behavioural findings. It is a reconstruction—I hope, a sympathetic reconstruction—of a proposal made by BPW.

## Bleichrodt et al.’s corrective procedure

BPW focus on the problem of constructing a measure of the utility of being in a given state of health over some given interval of time, using the stated preferences of some individual.

In the simplest version of this problem, there is a convex *reference set H* of health states over which the individual has a strict preference ranking. This set contains a uniquely best state *h*^+^ (full health) and a uniquely worst state *h*^–^ (death). We can define a function *u*(.) that assigns a real-valued utility index to each state and normalise it by setting *u*(*h*^–^) = 0 and *u*(*h*^+^) = 1. Consider any other state *x* in *H*. One standard method of identifying *u*(*x*) is by *probability equivalence*. We elicit the probability *p* such that the respondent is indifferent between *x* with certainty and the prospect, denoted (*h*^+^, *p*; *h*^–^, 1−*p*), that gives *h*^+^ with probability *p* and *h*^–^ otherwise; *p* is the *probability equivalent* of *x*, which can be written as *p* = PE(*x*). It is immediately obvious that if the respondent’s preferences have an expected utility (EU) representation, *u*(*x*) = *p*. Another standard method of identifying the utility function uses *certainty equivalence*. Given any probability *q*, we can define a prospect (*h*^+^, *q*; *h*^–^, 1−*q*). We elicit the state *y* in the reference set such that the respondent is indifferent between that prospect and *y* with certainty; *y* is the *certainty equivalent* of *q*, which can be written as *y* = CE(*q*). If the respondent’s preferences have an EU representation, we can infer that *u*(*y*) = *q*.

We can check the consistency of the two methods by taking a health state *w* ∈ *H*, finding its probability equivalent PE(*w*), and then finding the certainty equivalent of that probability, i.e., CE[PE(*w*)]. If the methods are consistent, we will find that *w* = CE[PE(*w*)]. Unfortunately, that is often not the case: the usual finding is that CE[PE(*w*)] > *w*. It is as if respondents are more risk-averse when converting outcomes to a probability scale than when converting probabilities to an outcome scale.

Another way of describing the discrepancy between probability equivalence and certainty equivalence is to say that we can often find pairs (*x*, *p*) such that PE(*x*) > *p* and CE(*p*) > *x*: when preferences are elicited using the PE frame, the respondent states a strict preference for the certainty of *x* over the uncertain prospect (*h*^+^, *p*; *h*^–^, 1−*p*), but when preferences are elicited using the CE frame, she states the opposite preference. If the relevant survey instruments state the value of *p* explicitly, and if the same descriptions of *h*^+^, *h*^–^ and *x* are used in both frames, it seems inescapable that the two frames are eliciting preferences between exactly the same pair of options. The problem is that those preferences are *context-dependent*.

BPW offer a psychological explanation of context dependence in relation to PE and CE. Following Hershey and Schoemaker (1985), they suggest that the certain outcome *x* in a PE survey is a ‘salient reference point’, while the uncertain prospect (*h*^+^, *p*; *h*^–^, 1−*p*) in a CE survey is not (p. 1503). CE respondents assign the difference between *x* and *h*^–^ to the domain of (actual or forgone) gains, while PE respondents assign it to the domain of losses. The CE–PE discrepancy is attributed to loss aversion.

The intuition behind this idea is more obvious in the case of lotteries with money outcomes. Suppose you unexpectedly win some competition. For your prize, you can choose between €100 for sure or a 50–50 lottery with outcomes of €0 and €250. One way of thinking about this choice is that the €100 and €250 are both gains relative to your prior expectations. Alternatively, you might think that you are now €100 better off than you were, and the issue is whether to incur a 50 per cent chance of losing €100 for a 50 per cent chance of gaining €150. Intuitively, taking the chance is more attractive in the first frame than in the second. To ask which frame is correct would be a category mistake: frames of this kind are neither correct nor incorrect, in the same way that Edgar Rubin’s famously ambiguous picture is neither correctly the profile of a vase nor correctly those of two human faces. If one way of thinking about the two prizes prompts you to choose the certainty and the other prompts you to choose the gamble, you might reasonably say your preferences over the two options are precise but frame-dependent; or you might reasonably say that they are imprecise in the sense of Butler and Loomes (2007).^2^ But, I maintain, it would be a mistake to ask which is your *true* preference and which is *biased*. On one possible reading, however, BPW claim the contrary.

Their response to the discrepancy between PE and CE is a ‘proposal for correcting biases’ (pp. 1504–1506). This *corrective procedure* works as follows. In the *descriptive* part of their analysis, BPW model actual decisions over lotteries that have well-specified outcomes and probabilities. For this purpose, they use a parametric form of prospect theory which corresponds in many respects both with the original version of that theory (Kahneman & Tversky, 1979) and with its later ‘cumulative’ version (Starmer & Sugden, 1989; Tversky & Kahneman, 1992). It has a function *u*(.) which assigns a utility value to each outcome, a parameter λ which defines the individual’s degree of loss aversion, and two parameters γ^+^ and γ^–^ which define the individual’s ‘probability transformation functions’ for gains and losses respectively. If λ = γ^+^  = γ^–^ = 1, the model reduces to EU. In a significant modification of Kahneman and Tversky’s theories, BPW define utility as a function of ‘final’ outcomes rather than as changes relative to a reference point, as is the case in prospect theory. In BPW’s model, reference points affect preferences only through the loss aversion parameter, thus enforcing a separation between utility and loss aversion. By fitting the descriptive model to actual choice data, they estimate the utility function and the descriptive values of λ, γ^+^ and γ^–^. They then convert this into a *prescriptive* model by keeping the estimated utility function but substituting λ = γ^+^  = γ^–^ = 1.

BPW are surprisingly brief in explaining the rationale of this procedure. They say that their ‘normative assumption’ is that EU is ‘the right normative model for decision under uncertainty’ (pp. 1498–1499). They say that they are assuming two ‘biases’ or ‘deviations from expected utility’—loss aversion and probability transformation—which result from normatively irrelevant psychological mechanisms. They explain that their treatment of loss aversion applies only to cases in which (as in the elicitation of CE and PE) the distinction between gain and loss is a pure framing effect, hence satisfying the condition of normative irrelevance. They do not add a similar qualification to their treatment of probability transformation, presumably because these transformations are interpreted as psychologically induced distortions of objective probability data; BPW are taking it be self-evident that only the objective data are normatively relevant. Defining the concept of bias and summarising their approach, they say:

The essence of the problem [to be addressed in their paper] lies in the biases, i.e., the discrepancies between elicited preferences and the true preferences according to a rational model in which these preferences are to be implemented. Observed inconsistencies prove that biases are present so that corrective procedures are called for. (pp. 1498–1500)

Substituting λ = 1 is treated as correcting loss aversion bias and substituting γ^+^  = γ^–^ = 1 is treated as correcting probability transformation bias.

For a present-day behavioural economist, reading BPW’s paper 20 years after it was written, it would be natural to interpret ‘bias’, ‘correction’ and ‘true preference’ in the ways that these concepts are used in the literature initiated by Camerer et al. (2003) and Sunstein and Thaler (2003). On that interpretation, BPW are claiming that respondents’ revealed preferences are incorrect relative to some standard that, in some reflective sense, is endorsed by the relevant respondent (or ‘client’). Interestingly, however, BPW do not interpret that incorrectness as a failure of rationality by the client: ‘We emphasize that biases and inconsistencies are not to be interpreted as irrationalities on the client’s part. Instead, they designate deficiencies in our measurement instruments that, even if the best currently available, do not tap perfectly into the clients’ values’ (p. 1500). BPW suggest that, in the absence of cost and practicability constraints, the best method of eliciting clients’ values is though ‘interactive sessions’ between interviewers and clients, in which ‘the client is asked to reconsider inconsistent choices’. BPW’s corrective procedure is recommended only as a ‘quick and dirty’ attempt to achieve the same results (p. 1499). Nevertheless, there seems to be an implicit assumption that each individual has true preferences (or ‘values’) that are consistent with EU and are capable of being elicited by a careful interviewer. Because low-cost survey instruments are subject to bias, survey responses do not directly reveal individuals’ true preferences. The objective of the corrective procedure is to retrieve true preferences, as accurately as possible, from survey responses. In the following two sections, I consider two alternative interpretations of BPW’s concept of ‘correction’.

## BPW’s corrective procedure interpreted as debiasing

In the second paragraph of their paper, BPW declare: ‘It is commonly assumed in decision analysis, and also in this paper, that the right normative model for decision under uncertainty is expected utility’ (pp. 1498–1499). They do not specify *for whom* it is the right model. In the contexts that BPW consider, stated preference studies are a link between two classes of agent—the *survey respondents* whose stated preferences provide the input to the analysis, and the *public decision-makers* who act on the output. My guess is that BPW believe it to be right for both types of agent, but my concern is with the assumptions that their approach requires. In this section, I investigate the implications of assuming that EU is the right normative model for survey respondents.

Consider BPW’s statement that observed inconsistencies prove the existence of biases and hence the need for corrective procedures. According to their account, an ‘observed inconsistency’ is a configuration of revealed preferences that contravenes the axioms of EU. Clearly, an individual who exhibits such an inconsistency is not acting on any of the preference orderings that satisfy those axioms. But there are many such preference orderings. If, as is the standard practice in economics and decision theory, EU is interpreted as an axiomatic theory of rational choice, it is an implication of that theory that a rational individual will act on *one of* those orderings; but there is nothing in the theory that determines *which* ordering that is. EU is not a theory about what makes one ordering more rational than another. Nor is it a theory of any process by which an individual might come to have consistent preferences. It is simply a theory about the properties *by virtue of which* preferences are mutually consistent. In other words: EU lacks any concept of *true* preference. Since bias is meaningful only in relation to some standard of correctness, EU also lacks any concept of bias.

Theorists often appeal to the intuitive reasonableness of the consistency properties that are expressed by the EU axioms. BPW may have some such thought in mind when they suggest that respondents would want to revise choices that an interviewer had shown to be inconsistent. But the most fundamental EU axiom, completeness, is not a consistency condition. In the canonical axiomatic statement of EU, Savage (1954, p. 21) recognises that people sometimes experience ‘introspective sensations of indecision or vacillation, which we may be reluctant to identify with indifference’, and justifies his completeness axiom only on pragmatic grounds. Consider a respondent who would choose *x* from the set {*x*, *y*} if the decision problem were presented in frame *F*, but *y* if it were presented in frame *G*. (Think of the difference between PE and CE questions.) An interviewer asks her to reconsider those choices. Suppose she replies: ‘I don’t have any firm preference (or indifference) between *x* and *y*. I know that the difference between *F* and *G* is just a matter of framing. But when I think about the options in frame *F*, I feel an inclination to choose *x*, and when I think about them in frame *G*, I feel an inclination to choose *y*. Why do I need to reconsider either of those choices?’^3^ BPW are entitled to declare that this response would be contrary to what they judge to be the right normative model, but that would not help the respondent to discover her true preference between *x* and *y*. Nor would it help a public decision-maker to reconstruct such a preference. I conclude that the supposed normativity of the EU axioms does not justify the claim that behaviour that contravenes those axioms is evidence of error or bias.

Another way of thinking about true preference is more empirical. If prospect theory and EU are interpreted descriptively, BPW’s version of prospect theory is a generalisation of EU in which explanatory factors can be separated into those that can be represented in an EU model and those that cannot. It is widely accepted that EU models fit observed choices at least moderately well, but that prospect theory models perform somewhat better, even after allowing for the fact that they have additional parameters. For example, Hey and Orme (1994) compare EU with a model closely related to cumulative prospect theory (‘Rank Dependence with the Power Weighting Function’) as rival explanations of a body of experimental data. On the basis of likelihood ratio tests, they conclude that the prospect theory model is among a set of generalisations of EU that perform significantly better than EU itself (pp. 1307–1312). The empirical success of prospect theory might suggest the interpretation that it represents how true preferences (the EU component of the theory) are distorted by biases (the non-EU component).

To pursue that idea, we need to ask why, as a matter of empirical psychology, EU works as well as it does. An obvious answer is that it picks up three effects that one would expect to have a strong influence on most people’s choices. The utility function picks up the effect that, other things being equal, an uncertain prospect is more likely to be chosen, the better are the outcomes to which it can lead. By taking account of probabilities, EU picks up the effect that, other things being equal, an uncertain prospect is more likely to be chosen, the higher the probabilities of its better outcomes and the lower the probabilities of its worse outcomes. By allowing the utility function to be non-linear, EU can pick up the effect that, when expected value is held constant, people tend to prefer less risky prospects to more risky ones. The EU representation combines these three effects in a simple functional form. If we ask why prospect theory performs better than EU, there is an equally obvious answer—that it takes account, not only of the psychological effects that are picked up by EU, but also of two others. One such effect, picked up by the parameter λ in BPW’s model, is loss aversion: other things being equal, outcomes evoke different psychological responses if they are perceived as gains than if they are perceived as losses. The other effect, picked up by the parameters γ^+^ and γ^–^, is a stimulus–response effect of diminishing sensitivity: other things being equal, people are more responsive to given increments of probability when probabilities are closer to the end-points of the scale on which probability is measured. If BPW’s corrective procedure is to be interpreted as debiasing, it seems that we must read BPW as claiming that the first three effects are properties of true preferences and that the other two are biases.

At some points in their paper, BPW make essentially this claim. Discussing loss aversion, they distinguish between (on the one hand) ‘intrinsic reasons’, ‘intrinsic utility’ and ‘the genuine von Neumann-Morgenstern utility function’ and (on the other) ‘irrelevant reframings’ (pp. 1500–1501). On one possible reading, BPW are interpreting intrinsic utility as hedonic experience, and defining an individual’s true preferences as those that are consistent with the maximisation of expected *experienced* utility (compare Kahneman et al., 1997). On that reading, the aim of BPW’s corrective procedure would be to identify experienced utility. BPW also report an experiment that elicited valuations of health states using PE and CE frames. They find that their corrective procedure greatly reduces the PE–CE discrepancy, and interpret this result as suggesting that ‘our corrective procedures are in the right direction and lead to a closer approximation of true utility’ (p. 1509). It seems to me that this result supports the empirical hypothesis that the discrepancy is a result of loss aversion, but any implications about true utility are determined by BPW’s definitions. At other points in the paper, however, BPW are more circumspect. For example, the suggestion that their corrections ‘lead to a closer approximation to true utility’ is qualified by: ‘There is no gold standard for utility, and such appropriateness claims for utility therefore have to be speculative. Our speculations are based on prospect theory’ (p. 1509).

## BPW’s corrective procedure interpreted as regularisation

In justifying their proposal, BPW are careful to avoid extravagant theoretical claims. They claim only that they know of no better way of dealing with an unavoidable problem:

We are well aware that many of the assumptions underlying our proposal are controversial, such as the very existence of true underlying preferences. These assumptions are, however, the best that we can think of in the current state of the art for situations where decisions have to be taken, as good as possible, on the basis of quick and dirty data.’ (p. 1500; see also p. 1510)

That *decisions have to be taken* is a characteristic feature of many applications of stated preference methods. Take the case of eliciting the utilities of health states. In health care systems funded through social insurance, it is a fundamental organising principle that health care is allocated according to patients’ medical needs rather than their willingness to pay as individuals. Physicians, hospital administrators and government officials have to take decisions about the allocation of limited resources between treatments or programmes that lead to different combinations of effects on people’s health states. Those decision-makers can reasonably ask health economists for guidance about the preferences of the citizens who are the potential users (and ultimate funders) of the health care system. BPW’s concern is with methods for giving that guidance. Their pragmatic stance is entirely appropriate.

In typical applications of stated preference methods, there is no one-to-one correspondence between the *practical* decision problems faced by public decision-makers and the *survey* decision problems in which preferences are elicited. For example, a PE or CE survey uses a small number of abstract choice problems to elicit respondents’ utility indices for generic health states. The respondents themselves are a representative sample of a much larger population of potential recipients of health care. The elicited indices might then be used to guide public decisions about the allocation of resources between the treatment of specific medical conditions experienced by specific (and perhaps non-overlapping) groups of patients. Unavoidably, the contextual differences between survey and practical problems are far larger than the differences between alternative survey designs that induce context-dependent responses. The connection between practical problem and survey must be made by some theoretical model.

This thought suggests a different reading of BPW’s claim that EU is the right normative model for their enterprise. Their essential assumption may be that EU is the right model for *public decision-makers* to use when drawing policy inferences from survey responses. Recall BPW’s definition of ‘bias’ as a discrepancy between elicited preferences and ‘true preferences according to a rational model in which these preferences are to be implemented’. Notice that the ‘rational model’ here is not a model of the respondent’s psychology or behaviour; it is the model that is to be used by the public decision-maker when acting on his interpretation of the survey data. The implication seems to be that ‘true preferences’ are true according to the rationality of the decision-maker, not that of the respondent.

An immediate reason for thinking that this is the right line for BPW to take is that their paper is addressed to public decision-makers, either directly or through the mediation of other health economists. Health economists can be expected to concur with the standard assumptions of decision analysis. Although that is less likely to be true of public decision-makers, health economists are entitled to base their advice on established principles of decision analysis, and where necessary to include explanations of those principles in that advice. In contrast, most survey respondents know nothing about formal decision analysis, and it would be contrary to good survey methodology to preface questions with normative assertions about the rationality or irrationality of possible responses.

A more fundamental reason stems from the fact that (as I pointed out in Sect. 2), EU does not tell anyone what decision they should make in any particular case; it is a theory of consistency between an agent’s responses to different decision problems. Survey respondents, and private citizens more generally, are entitled to say that they don’t care whether or not their decisions are mutually consistent—that they are happy to take one choice at a time, acting on whatever seems important to them at the time. In contrast, public decision-makers are responsible to the citizens on whose behalf they act. They can reasonably be expected to be able to give consistent justifications for their decisions. It is a standard assumption of welfare economics that such justifications should refer to individuals’ welfares. Thus, we (and, more importantly, citizens) might expect a public decision-maker to act on a consistent model of each individual’s welfare. If a public decision-maker believes that EU is the right normative model for him to use, he needs to use a model of each individual’s welfare that satisfies the EU axioms—that is, in which the relation ‘*x* gives at least as much welfare as *y* to person *i*’ has the same formal properties as weak preference in an EU model. He can recognise the need for such a model of welfare without claiming that the model corrects errors in individuals’ choices.

## Two specifications of prospect theory

In considering how best to interpret BPW’s corrective procedure, it is illuminating to look at their specification of prospect theory. BPW use a particular form of prospect theory that is based on, but significantly different from, the theory proposed by Tversky and Kahneman (1992: henceforth, TK). Since both versions use essentially the same model of cumulative probability transformation, I focus on their respective treatments of reference points. For the purposes of this comparison, I define the domain of prospect theory as the set of lotteries of the form (**x**, **p**) = (*x*_1_, *p*_1_; …; *x*_*m*_, *p*_*m*_), where each *p*_*i*_ is a probability such that ∑_*i*_* p*_*i*_ = 1, and each *x*_*i*_ is a level of final wealth, measured in money units. One wealth level, *r*, is the constant reference point.

In BPW’s model, a utility function *u*(.) assigns a real-valued index of *intrinsic utility* to each level of wealth, independently of the reference point. BPW also have a concept of *reference-dependent utility*, which can be represented by a function u*(*.* | *r*); u*(*x* | *r*) is interpreted as the utility of *x*, viewed in relation to the reference point *r*. This function is specified as:

1 $$\begin{gathered} u^*\left( {x|r} \right) \, = u\left( r \right) \, + \, \left[ {u\left( x \right) \, {-}u\left( r \right)} \right] \, = u\left( x \right)\ {\text{if}}\quad x \ge r, \,{\text{and}} \hfill \\ u^*\left( {x|r} \right) \, = u\left( r \right) \, {-}{\lambda}\left[ {u\left( r \right) \, {-}u\left( x \right)} \right] \, = {\lambda}u\left( x \right) \, {-} \, ({\lambda}{-}{ 1})u\left( r \right)\ {\text{if}}\quad x \le r, \hfill \\ {\text{with}}\,\lambda \ge {1}. \hfill \\ \end{gathered} $$

Preferences over lotteries, conditional on the reference point *r*, are represented by the function

2 $$ U\left( {{\mathbf{x}},{\mathbf{p}}|r} \right) \, = {\sum \nolimits_i} {\pi_i} {u^*}\left( {x_{i} |r} \right), $$

where π_*i*_ is the *decision weight* associated with *x*_*i*_. The vector of decision weights **π** is determined cumulatively from (**x**,** p**) using a probability transformation function, but the details of this are not relevant here. Notice that, if λ = 1 and π = p, *U*(**x**,** p** | *r*) = ∑_*i*_π_*i*_* u*(*x*_*i*_) for all *r*, and so the model reduces to EU.

A significant feature of this model is that attitudes that relate to reference points (i.e., loss aversion and probability transformation) are defined independently of intrinsic utility. This feature greatly simplifies the problem of eliciting reference-independent utility indices. It is also compatible with the debiasing interpretation of the corrective procedure if (as I suggested as a possible reading of BPW in Sect. 2), BPW are equating intrinsic utility with experienced utility, and are treating reference-dependence effects as biases in individual decision making. However, this feature comes at what might be viewed as a cost: BPW’s model does not take account of one of the main psychological mechanisms of TK’s prospect theory. Let me explain.

According to TK, prospect theory rests on two fundamental properties of human psychology, both of which involve reference-dependence: loss aversion and diminishing sensitivity. Diminishing sensitivity explains both the inverse-S shape of the probability transformation function and the properties of the ‘value function’—the analogue of the utility function in EU (TK: 302–303). In TK’s model, *value* is defined as a function of gains and losses of wealth, measured relative to a reference point. Formally, a function *v*(.) assigns a real-valued index of value to each increment of wealth *x*−*r*. In the parametric form of the model, this function is specified as:^4^

3 $$ \begin{gathered} v\left( {x{-}r} \right) \, = \, \left( {x{-}r} \right)^{{\alpha}}\ {\text{if}}\quad x \ge r,{\text{ and}} \hfill \\ v\left( {x{-}r} \right) \, = \, {-}{\lambda}\left( {r{-}x} \right)^{{\alpha}}\ {\text{if}}\quad x \le r, \hfill \\ {\text{with }}0 \, < \alpha {\text{ and}}\,\lambda \ge {1} \hfill \\ \end{gathered} $$

If α < 1, lower values of α represent greater degrees of diminishing sensitivity to gains and losses. Preferences over ‘prospects’ in TK’s sense of the term (i.e., lotteries defined in terms of gains and losses of wealth), are represented by the function

4 $$ V\left( {{\mathbf x}{-}r, {\mathbf p}} \right) \, =  \sum\nolimits_{i} {\pi _{i} }    v\left( {x_{i} {-}r} \right), $$

with π_*i*_ defined as in BPW’s model. TK’s model is not a generalisation of EU, but the two theories share a common special case: if α = λ = 1 and** π** = **p**, TK’s model is formally equivalent to an EU model in which *u*(*x*) = *x* for all *x* (i.e., in which preferences are risk-neutral).

In their original statement of prospect theory, Kahneman and Tversky (1979: 277–278) say: ‘Strictly speaking, value should be treated as a function in two arguments: the asset position that serves as reference point, and the magnitude of the change (positive or negative) from that reference point’. They do not develop this idea in any detail, but suggest that, for greater predictive accuracy, the value function should be assumed to be more linear (i.e., to have an α parameter closer to 1) at higher values of *r*. However, they point out that, within the range of values that has been studied experimentally, this effect has been found to be very small. Thus, treating value as a function only of increments of wealth ‘generally provides a satisfactory approximation’.

It seems that TK have in mind a more general version of prospect theory in which there is some concept of intrinsic utility. An obvious way of generalising the official version of prospect theory would be to define a function *u*(.) which assigns a real-valued index of intrinsic utility to each level of wealth, and then to substitute intrinsic utilities for wealth levels in expressions (3) and (4). TK would presumably assume diminishing marginal intrinsic utility in this general model, but would treat linearity of intrinsic utility as a satisfactory approximation when gains and losses are modest. In such a model, loss aversion, probability transformation and diminishing sensitivity to gains and losses would tend to induce deviations from EU, but diminishing marginal intrinsic utility would not. Since Kahneman and Tversky (1979: 277) interpret deviations from EU as leading to ‘normatively unacceptable consequences’ that decision makers would want to ‘correct’, it is reasonable to infer that, for TK, loss aversion, probability transformation and diminishing sensitivity to gains and losses are all biases. Notice the implication that, when gains and losses are modest, risk aversion is almost entirely attributable to factors that TK would treat as biases: attitudes to risk do not convey information about intrinsic utility. Rabin (2000) provides theoretical support for this conclusion by showing that an EU model that is calibrated on data from choices between small- or medium-scale lotteries would imply implausibly strong risk aversion in decisions with higher stakes. Rabin suggests that this anomaly can be explained by loss aversion.

I now return to BPW’s analysis. It is fundamental to BPW’s method of eliciting utilities from lottery choices that respondents’ attitudes to risk *do* convey information about intrinsic utility. Of course, BPW are analysing responses to hypothetical decisions in which gains and losses are *not* modest: they are matters of life and death. Nevertheless, their model differs from TK’s by not taking account of a mechanism of diminishing sensitivity which might be expected to inflate observed risk aversion.

BPW justify this modification on pragmatic grounds, as appropriate for their project of eliciting respondents’ utility functions from survey data. They note that in TK’s model, loss aversion is ‘incorporated in the utility function’. In contrast: ‘We separate loss aversion and utility because we will consider varying reference points and because we want to establish a link with expected utility. Therefore, our utility function [*u*(.) in my notation] describes an intrinsic utility of final wealth. … [W]e do not assume the particular shape of the value function suggested by prospect theory, the purpose of our study being to measure general utility functions’ (p. 1501). If BPW’s project is one of regularisation, this is reasonable enough: in choosing which of two descriptive models to use, they are favouring the one that is more easily regularised into the kind of normative model that the planner needs. But this kind of pragmatic justification would be out of place if the objective were to retrieve citizens’ true preferences by correcting biases in their decisions.

## Regularisation as a general method

In this section, I offer a general representation of a procedure of regularisation that is formally consistent with, and would be defensible as a justification of, BPW’s corrective procedure.

Consider a given public decision-maker (for short, the *planner*, him) who is making judgements about the welfare of some individual (the *citizen*, her), given some information about how the citizen would choose in various situations.

The planner selects a *descriptive theory D* of individual preferences over some domain *O* of possible outcomes for an individual. In BPW’s analysis, *D* is their version of cumulative prospect theory. Because I am focusing on applications of prospect theory, I treat preference as a reference-dependent relation: for any *x*, *y*, *r* ∈ *O*, *x* ≽ *y* | *r* denotes ‘*x* is weakly preferred to *y*, viewed from the reference point *r*’. *D* imposes various conditions on preferences (for example, that they are weakly loss averse). The set of alternative preference relations that satisfy those conditions is *P*(*D*). In the planner’s judgement, these conditions represent empirical regularities which, to a reasonable approximation, are true of the citizen’s actual decisions in the contexts in which the planner’s welfare judgements will be applied. Notice, however, that *D* is a *theoretical model of* the citizen’s psychology which the planner chooses to use; it is not the psychology itself.

The planner also endorses a *normative theory N* of reference-dependent preferences (defined as before) over *O*. In BPW’s analysis, this theory is EU. *N* imposes some conditions on preferences; the set of alternative preference relations that satisfy those conditions is *P*(*N*). Note that one possible condition, implicit in EU, is that preferences are not affected by reference points (i.e., *x* ≽ *y* | *r*′ ⇔ *x* ≽ *y* | *r*″ for all *x*, *y*, *r*′, *r*″ ∈ *O*). The planner endorses these conditions as principles of rationality that ought to be satisfied by public judgements about individual welfare.

As the final component of this scheme, the planner chooses a *regularisation function* ρ: *P*(*D*) → *P*(*N*). Consider any (reference-dependent) preference relation ≽_*D*_ ∈ *P*(*D*). This relation is a potential description of the actual preferences of the citizen. ρ(≽_*D*_), the *regularisation* of ≽_*D*_, is the preference relation which, as judged by the planner, best represents the ordinal properties of that citizen’s welfare, given the consistency conditions that the planner accepts as normative requirements on his judgements.

The triple (*D*, *N*, ρ) represents the planner’s understanding of what he is doing. Each of the three elements of this triple embodies judgements made by the planner. Although the planner is constrained by known facts about the citizen’s preferences, those facts do not fully determine which model of preference should be used as *D*. One legitimate criterion for choosing between alternative descriptive models is the ease with which each model can be regularised. For any given *D*, the regularisation function embodies the planner’s judgements about the relative importance of preserving different properties of citizens’ actual preferences. *N* embodies the planner’s normative judgements about the rationality conditions that his judgements should satisfy.

Now consider the following two formal conditions on (*D*, *N*, ρ), stating relationships between *D* and *N* that might be seen as desirable:

*Inclusion (of Consistent Preferences)*. *P*(*N*) ⊆ *P*(*D*).

*Conservation (of Consistent Preferences)*. For all preference relations ≽ ∈ *P*(*D*): [≽ ∈ *P*(*N*)] ⇒ [ρ(≽) = ≽].

Inclusion is satisfied if the descriptive theory is a generalisation of the normative theory—that is, if the empirical restrictions imposed by the descriptive theory do not exclude preference relations that the normative theory deems to be consistent. Conservation is satisfied if the regularisation function does not alter descriptive preference relations that already satisfy the rationality conditions of the normative theory.

BPW’s analysis illustrates a general method for constructing (*D*, *N*, ρ) triples that satisfy Inclusion and Conservation. This method starts from a descriptive model in which a suitably normalised parametric function represents descriptive preferences over the relevant domain *O*. The parameters of this model can be indexed by *j* = 1, …, *k*; specific parameter values are denoted by *a*_*j*_. Restrictions on parameter values are specified; these determine *P*(*D*). The parameters of the descriptive model are of two types—*core* (*j* = 1, …, *l*) and *non-core* (*j* = *l* + 1, …, *k*). For each non-core parameter *j*, a *regular* value *a*_j_* is specified. For each vector **a** of parameter values, the regularisation of that vector is defined by *g*(**a**) = (*a*_1_, …, *a*_*l*_, *a*_*l* + 1_*, …, *a*_k_*). The specification of regular values must be such that, for every **a** that satisfies the restrictions imposed by the descriptive theory, *g*(**a**) satisfies the same restrictions. Thus, regularisation defines a special case of the descriptive theory, inducing a subset of *P*(*D*). *N* is then defined as that special case; ρ is defined so that it describes this process of regularisation. This ensures that Inclusion and Conservation are both satisfied. In BPW’s analysis, the descriptive model is their version of prospect theory. The core parameters of this model specify the utility function. The non-core parameters are the loss aversion parameter λ and the probability transformation parameters γ^+^ and γ^–^; for each of these parameters, the regular value is 1. The regularisation induces EU as the special case.

As I have described this method, ≽_*D*_ is a model of the citizen’s actual preferences, as revealed in her decisions. ρ(≽_*D*_) states the planner’s judgements about the citizen’s welfare. For each *j* ∈ {*l* + 1, …, *k*}, *a*_j_* – *a*_*j*_ is an adjustment that is used to ensure that those judgements are compatible with *N*, the theory of rationality that planner endorses. There need be no claim that each *a*_*j*_ – *a*_j_* represents a bias or error on the part of the citizen, nor that ρ(≽_*D*_) is a model of the citizen’s true preferences.

I have presented this method—the method of *structural regularisation*—as *a* defensible procedure by which a planner might transform a citizen’s revealed preferences into a welfare ranking that satisfies the principles of rational consistency that the planner endorses.^5^ I do not claim that it is the only defensible form of regularisation. An obvious alternative is *direct regularisation*: instead of estimating a descriptive model and then transforming its parameters, one might simply estimate a parameterised version of the normative model using the citizen’s revealed preferences as data—for example, by fitting an EU model to the decisions of a citizen whose behaviour is better explained by BPW’s version of prospect theory. By assumption, the EU model would be misspecified, but it might be a better fit to the citizen’s revealed preferences than the regularised model produced by BPW’s corrective procedure. The relative merits of different forms of regularisation is an important topic, but beyond the scope of the current paper.

## Implications for behavioural welfare economics

Ever since the influential papers of Camerer et al. (2003) and Sunstein and Thaler (2003), most attempts to use behavioural economics as a basis for policy guidance have followed the approach of debiasing rather than that of regularisation. Individual behaviour has been theorised as resulting from the interaction of true preferences and biases. The findings of behavioural economics have been interpreted as showing that individuals often make choices that do not reflect their true preferences. Such choices are classified as errors. Policy interventions are proposed with the aim of helping individuals to avoid these errors and counteracting the effects of errors that still occur. In this final section, which draws on arguments presented in more detail by Infante et al. (2016) and Sugden (2018), I suggest that behavioural economics has taken a wrong path.^6^ Regularisation, in the sense that I have characterised, is a coherent response to behavioural findings, but the claim that economics or behavioural science can distinguish between true preferences and biases is deeply problematic.

Most behavioural economists characterise their approach as grounded in empirical science, but the concepts of true preference and bias are not empirical. Viewed in the perspective of empirical psychology, preference is a mental state. In empirical economics, preference is usually interpreted either as a mental state that reveals itself in a person’s choices, or as a compact description of a regularity in a person’s choice behaviour. Mental states and acts of choice can *exist* or *occur*, but they can be neither true nor false, correct nor incorrect. Since the concept of bias is meaningful only in relation to some standard of correctness, the concept of a biased preference or biased choice is non-empirical too. Most of the empirical phenomena that behavioural economists have come to call ‘biases’ can be described more accurately as cases of *context-dependent preference*. Relative to a specific definition of the domain (for example, lotteries with specified probabilities and final outcomes) and to a specific definition of ‘context’ or ‘frame’ (for example, in terms of reference points), there is context dependence if a person’s preference between given objects in that domain varies according to the context in which those objects are presented. There is a *regularity* of context-dependent preference if, in some general class of situations, changes of context have systematic effects on preferences (for example, preferences over given objects are more risk-averse when those objects are presented as gains than when they are presented as losses). The research programme of behavioural economics has greatly increased the stock of knowledge about such regularities, but it is misleading to say (as many commentators do) that this work has discovered systematic biases in people’s preferences or choices.^7^

In many cases, individuals’ preferences are systematically dependent on contextual features that seem have no relevance for individuals’ welfare or interests. Behavioural economists often treat such observations as evidence of error. For example, Camerer et al. (2003: 1216–1217) describe one body of work in behavioural economics as showing that (what in 2003 were) standard models of preference fail to take account of factors that truly matter to individuals; behavioural economists build these factors into richer models of rational preferences. Camerer et al. then move straight to the ‘large part of behavioral economics [that] describes ways people sometimes fail to behave in their own best interests’ and therefore ‘create a need for paternalism’. But (as I argued in Sect. 2) inconsistency—the fact that a person’s choices cannot be rationalised by any plausible preference ordering—is not the same thing as error. Inconsistency does not imply the existence of a true preference ordering that behavioural economics can retrieve.

Of course, individuals’ beliefs about matters of fact can be correct (i.e., consistent with actual facts) or incorrect. It is sometimes proposed that preferences should be classified as mistakes if they are based on false or incorrectly formed beliefs about the relevant options. For example, Bernheim (2016: 48) defines a choice as mistaken if it is ‘predicated on a characterization of the available options and the outcomes they imply that is inconsistent with the information available to the decision maker’ and if ‘there is some other option in the opportunity set that the decision maker would select over the mistakenly chosen one in settings where characterisation failure does not occur’. But the ‘predicated on …’ clause requires us to be able to retrieve a description of the options that the individual has in fact used in arriving at her decision, and the ‘some other option …’ clause requires that we can identify a frame in which she acts on the correct descriptions of options. Unless the mental operations that lead to choices are sufficiently similar to those of rational-choice models, these requirements may be ill-defined. For example, consider probability transformations in prospect theory. It is tautological that any such transformation (other than the identity function) transforms true probabilities into false ones. But if prospect theory (with non-identity transformations) has a good fit to an individual’s choices between lotteries, can we infer that that individual acts on a mistaken characterisation of lotteries? I think not. In many of the experiments that have been used to estimate probability transformations, lotteries are explicitly and transparently described in terms of objective probabilities and monetary outcomes (e.g., Tversky & Kahneman, 1992). The subjects in these experiments clearly know the true probabilities, but, *viewed through the lens of prospect theory*, they behave as if they are mischaracterising them.

I suggest that much of what behavioural economists call ‘debiasing’, ‘error correction’ or the retrieval of ‘true preferences’ is more accurately described in terms of the model of regularisation I sketched in Sect. 5. The regularisation approach does not need to attribute the concepts of bias, error and true preference to individuals; its model of the individual is entirely descriptive. Normativity enters the analysis explicitly, through principles of rationality that are endorsed by the public decision-maker or ‘planner’. The planner claims only to have found a context-independent welfare ranking for each individual that is a satisfactory approximation to that individual’s revealed (and typically context-dependent) preferences, given his (the planner’s) conception of rational consistency. There is no claim to have uncovered any deeper truths than this.

Is anything lost by using this sparser conceptual framework? In the applications that motivated BPW’s analysis, the answer is ‘very little’. In these applications, there are public decisions *that have to be taken*. The public decision-maker who follows the regularisation approach can say that he has done his best to make rationally consistent decisions that are in accord with what he knows about citizens’ actual preferences. As BPW say, there is no gold standard here. And the decision-maker has a good answer to at least one of the ‘methodological concerns’ that BPW acknowledge as possible criticisms of their approach—the assumption that true preferences exist (p. 1510). The ‘decisions have to be taken’ condition encompasses many public decisions about the provision of public goods and the regulation of externalities, as well as BPW’s leading example, the setting of priorities in socialised health care systems. The regularisation approach might reasonably be applied also to many situations in which individuals delegate complex or burdensome decisions to professional specialists.^8^ For example, consider the relationship between a financial adviser and a client. The adviser’s task is to recommend a portfolio of assets into which the client will invest her savings. The client might reasonably expect those recommendations to be based on a consistent model of her long-term interests that reflects her personal attitudes to risk—but without having much idea about what that model would be. The adviser might use a risk tolerance questionnaire to elicit expressions of those attitudes and then regularise them into an EU-based model.

But what about the applications that motivated Camerer et al.’s and Sunstein and Thaler’s manifestos for ‘asymmetric’ and ‘libertarian’ paternalism, and which have featured so prominently in subsequent behavioural economics—applications in which public decision-makers supposedly help individuals to avoid erroneous choices in their private lives? Nothing in the regularisation approach debars a public decision-maker from using his regularisation of individuals’ preferences as the criterion for deciding when to try (whether by nudges or stronger measures) to influence individuals’ private decisions. What *is* disallowed by that approach is the claim that such interventions counteract biases and errors, and are in accord with individuals’ true preferences. Public decision-makers who want to be paternalistic merely need to be open about what they are doing.

## Footnotes

1 While accepting regularisation as a defensible approach, I favour a more radical response to behavioural findings, which uses opportunity rather than welfare as a normative criterion: see Sugden (2018).

2 Butler and Loomes offer an analysis of preference imprecision in relation to certainty equivalence and probability equivalence, applied to the preference reversal phenomenon.

3 It is not a good answer to appeal to the possibility of ‘money pumps’. Money pump arguments demonstrate undesirable consequences of *consistently* acting on preferences that violate specific axioms, not of not having preferences at all. See Cubitt and Sugden (2001) for a general analysis of what such arguments can and cannot achieve.

4 The general statement of TK’s model allows the exponent of the value function to be different for gains and losses but, in the light of experimental results, TK conclude that it is a satisfactory approximation to assume a common exponent (pp. 311, 316).

5 This paragraph builds on comments from an anonymous reviewer. The term ‘structural’ comes from Beshears et al., (2008: 1790–1791), who describe a formally similar procedure as ‘structural estimation’.

6 Rizzo and Whitman (2020) develop a similar critique of the concept of true preference that is used in behavioural welfare economics.

7 For example, Sunstein (2021) proposes a version of behavioural welfare economics that he calls ‘Hayekian’ on the grounds that it is ‘respectful of [Friedrich Hayek’s] most fundamental concerns’. To counter the objection that this proposal is much more paternalistic than Hayek’s actual writings, Sunstein argues that Hayek would have reached different conclusions if he had been aware of ‘modern behavioural findings about human error’, such as the prevalence of ‘present bias’, ‘optimistic bias’ and ‘availability bias’.

8 I thank an anonymous reviewer for suggesting this idea.
